# The *in vitro* and *vivo* anti-tumor effects and molecular mechanisms of suberoylanilide hydroxamic acid (SAHA) and MG132 on the aggressive phenotypes of gastric cancer cells

**DOI:** 10.18632/oncotarget.10643

**Published:** 2016-07-18

**Authors:** Hang Lu, Xue-feng Yang, Xiao-qing Tian, Shou-long Tang, Lian-qian Li, Shuang Zhao, Hua-chuan Zheng

**Affiliations:** ^1^ Cancer Center, The Key Laboratory of Brain and Spinal Cord Injury of Liaoning Province, and Laboratory Animal Center, The First Affiliated Hospital of Jinzhou Medical University, Jinzhou, China; ^2^ Department of Surgery, Panjin Central Hospital, Panjin, China; ^3^ Life Science Institute of Jinzhou Medical University, Jinzhou, China

**Keywords:** gastric cancer, suberoylanilide hydroxamic acid, MG132, aggressive phenotypes, chemotherapy

## Abstract

Here, we found that both SAHA and MG132 synergistically inhibited proliferation, glycolysis and mitochondrial oxidization, induced cell cycle arrest and apoptosis in MGC-803 and MKN28 cells. SAHA increased cell migration and invasionat a low concentration. SAHA induced the overexpression of acetyl histone 3 and 4, which were recruited to *p21*, *p27*, *Cyclin D1*, *c-myc* and *nanog* promoters to transcriptionally up-regulate the former two and down-regulate the latter three. The expression of acetyl-histone 3 and 4 was increased during gastric carcinogenesis and positively correlated with cancer differentiation. SAHA and MG132 exposure suppressed tumor growth by inhibiting proliferation and inducing apoptosis in nude mice, increased serum ALT and AST levels and decreased hemaglobin level, white blood cell and neutrophil numbers. These data indicated that SAHA and MG132 *in vivo* and *vitro* synergistically induced cytotoxicity and apoptosis, suppressed proliferation, growth, migration and invasion of gastric cancer cells. Therefore, they might potentially be employed as chemotherapeutic agents if the hepatic injury and the killing effects of peripheral blood cells are avoided or ameliorated.

## INTRODUCTION

Histone deacetylases (HDAC) function together with histone acetyltransferases (HAT) to accurately control the gene expression by altering nucleosome conformation, and the stability of several large transcription factor complexes. HDAC inhibitors are a promising class of anticancer epigenetic drugs to suppress growth, induce differentiation and apoptosis in cancer cells *in vitro* and *in vivo* [1, 2].

Suberoylanilide hydroxamic acid (SAHA, vorinostat) is a synthetic hydroxamic acid that inhibits class I and II HDACs via the coordination of its hydroxamic acid group with a zinc atom at the bottom of the catalytic cavity, and finally acetylates the histones within transcription factors [3, 4]. Actually, SAHA is approved by US Food and Drug Administration (FDA) and limitedly applied for solid tumors [5]. Reportedly, SAHA acts directly on the promoter region of the thioredoxin (TRx) binding protein-2 (TBP-2) gene and up- regulates TBP-2 expression. TBP-2 protein interacts with TRx protein, which inactivates such biological functions as scavenging reactive oxygen species (ROS) and activating ribonucleotide reductases [6, 7]. You et al. [8] demonstrated that SAHA inhibited the growth of HeLa cells, and induced their apoptosis, which was accompanied by PARP cleavage, caspase-3 activation, loss of mitochondrial membrane potential, and ROS production. Ding et al. [9] found that SAHA triggered MET and Akt phosphorylation in an HGF- independent manner. siRNA silencing of MET enhanced SAHA to induce the apoptosis of PC3 and A549 cells. Liu et al. [10] reported that SAHA inhibited the growth, reduced the migration and induced cell-cycle arrest, apoptosis and autophagy of paclitaxel-resistant ovarian cancer OC3/P cells.

Gastric cancer continues to be one of the deadliest cancers in the world and therefore the identification of new target drugs is thus of significant importance [11]. Yoo et al. [12] demonstrated that three-weekly SAHA-cisplatin regimen was feasible and recommended for further development in advanced gastric cancer. Zhou et al. [13] found that SAHA *in vitro* and *vivo* enhanced the antitumor activity of oxaliplatin by reversing the oxaliplatin-induced Src activation, increasing γH2AX expression, the cleavage of Caspase-3 and PARP in gastric cancer cells. Huang et al. [14] reported that RUNX3 was up-regulated by SAHA and increased the SAHA chemosensitivity in gastric cancer cells. Here, we observed the effects of SAHA and/or MG132 (a proteosome inhibitor) on the phenotypes of gastric cancer cells and its synergistic effects and subsequently clarified the related molecular mechanisms. To clarify the clinicopathological significance of acetyl-histones 3 and 4, their expressions were determined in gastric cancer and non-neoplastic mucosa (NNM) by western blot or immunohistochemisty, and compared with clinicopathological parameters of gastric cancers. Finally, their inhibitory effect on tumor growth was determined in tumor-bearing nude model.

## RESULTS

### The effects of SAHA and MG132 on the phenotypes of gastric cancer cells

**Figure 1:**
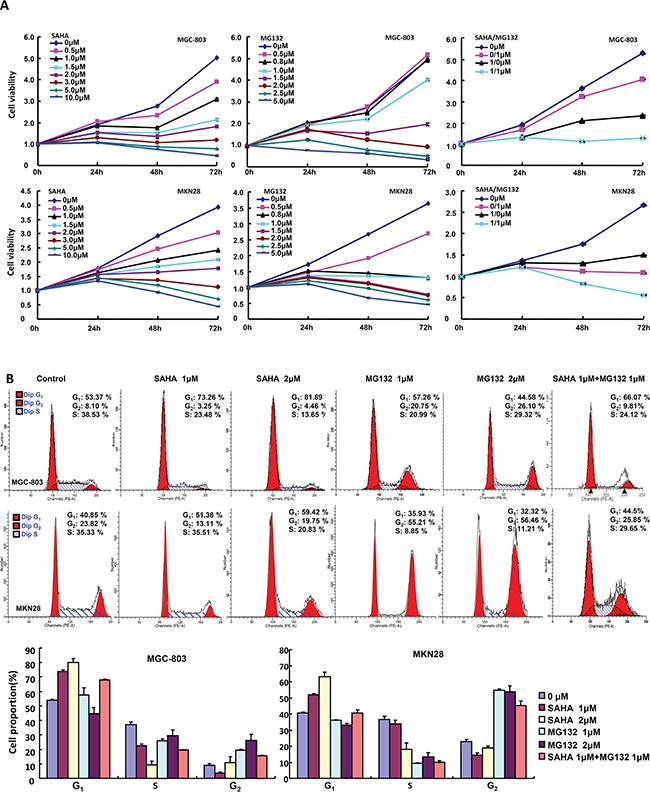
The effects of SAHA and MG132 on the proliferation of gastric cancer cells CCK-8 cell proliferation assays showed that SAHA and MG132 treatments induced cell death of MGC-803 and MKN28 cells in both concentration- and time-dependent manners with a synergistic effect for their combination **A.** Flow cytometric analyses after PI staining demonstrated that SAHA treatment induced G_1_ arrest; but MG132 did G_2_ arrest in both gastric cancer cells **B.**

**Figure 2:**
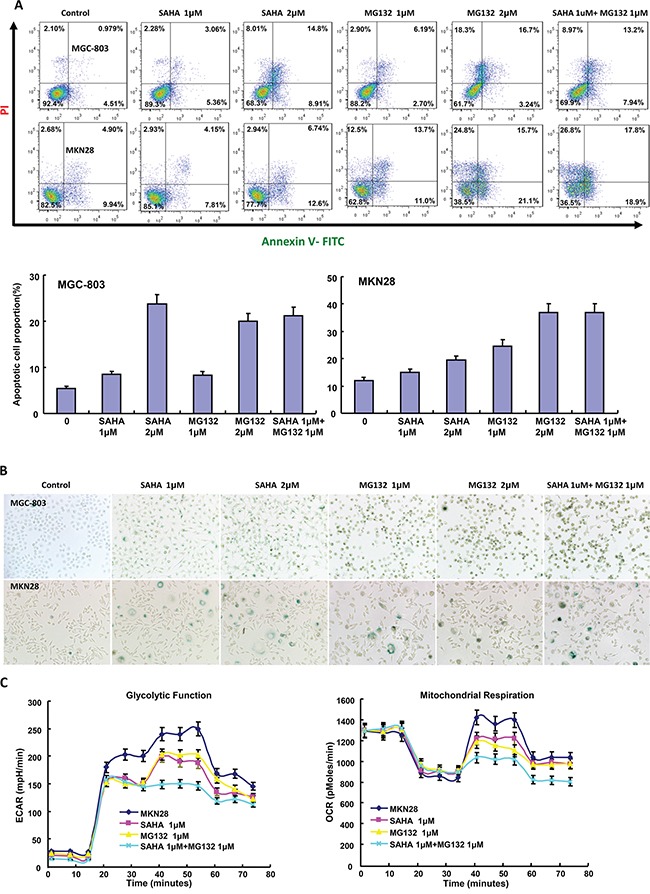
The effects of SAHA on apoptosis, senescence and glucose catabolism of gastric cancer cells After the exposure to SAHA and MG132, there appeared a high apoptotic **A.** and senescence **B.** level in MGC-803 and MKN28 in comparison to the control by Annexin V or β-galactoside staining respectively. Both drugs had a synergistic induction of apoptosis and senescence (**A** and **B**). Cellular energy metabolism assay was performed after the treatment of MKN28 cells with SAHA and/or MG132 **C.**

**Figure 3:**
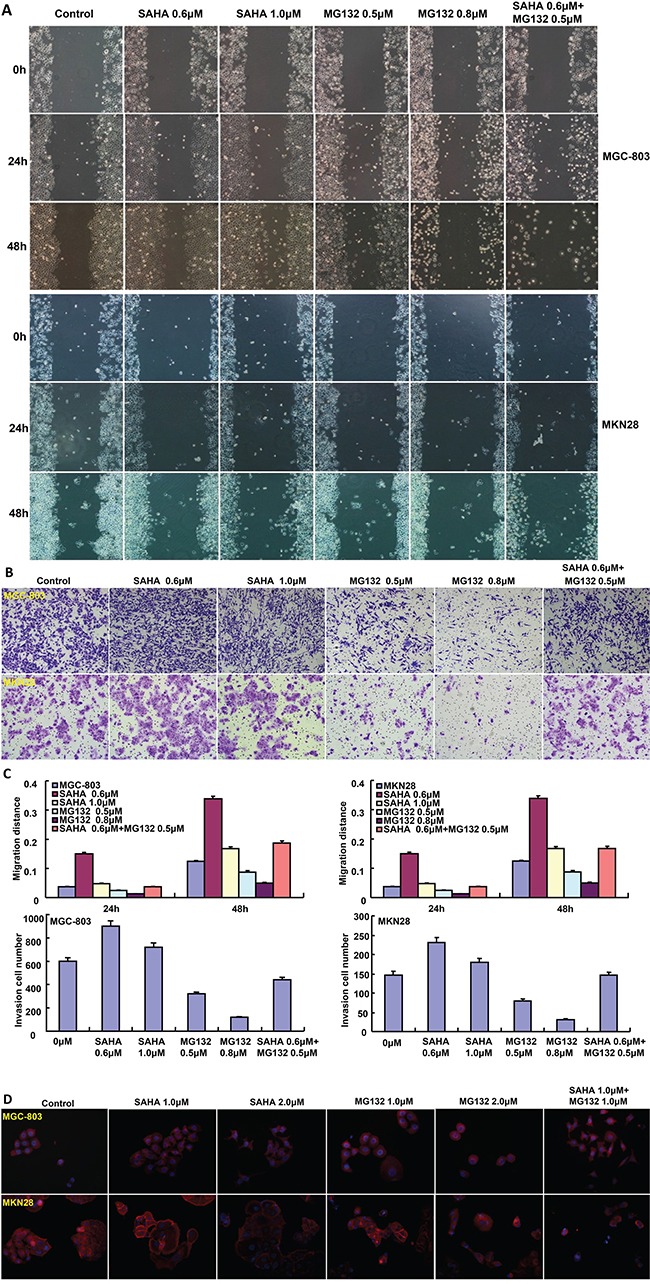
The effects of SAHA on migration and invasion of gastric cancer cells SAHA treatment increased the ability of MGC-803 and MKN28 cells to migrate and invade, especially at a low concentration (μM) by wound healing and transwell chamber **A-C.** MG132 exposure had an inhibitory effect on the migration and invasion of both cancer cells (A-C). The lamellipodia formation was observed using F-actin immunofluorescence staining after the treatment of both drugs **D.**

The exposure to SAHA and MG132 suppressed the proliferation of MGC-803 and MKN28 in both concentration- and time-dependent manners with a synergistic effect (Figure 1A, p<0.05). According to PI staining, SAHA treatment induced G_1_ arrest, while MG132 induced G_2_/M arrest in MGC-803 and MKN28 cells (Figure 1B). SAHA could reciprocally weaken the effects of MG132 on cell cycle. As shown in Figure 2A, the treatment with either SAHA or MG132 induced the apoptosis of MGC-803 and MKN28 cells in either concentration- dependent or synergistic manner according to Annexin-V and PI staining. It was the same for senescence, evidenced by β-galactosidase staining (Figure 2B). SAHA and MG132 synergistically suppressed glycolysis and mitochondrial respiration of MKN28 cells (Figure 2C, p<0.05). Wound healing and matrigel transwell invasion assays indicated that SAHA increased cell migration and invasion at a low concentration. MG132 suppressed the ability of gastric cancer cells to migrate and invade. MG132 ameliorated the effects of SAHA (0.6μM) on migration and invasion of gastric cancer cells (Figure 3A-3C). As shown in Figure 3D, 2.0μM SAHA relieved the lamellipodia formation in gastric cancer cells, while MG132 didn't.

### The molecular mechanisms about the reversing effects of SAHA or/and MG132 on the aggressive phenotypes of gastric cancer cells

**Figure 4:**
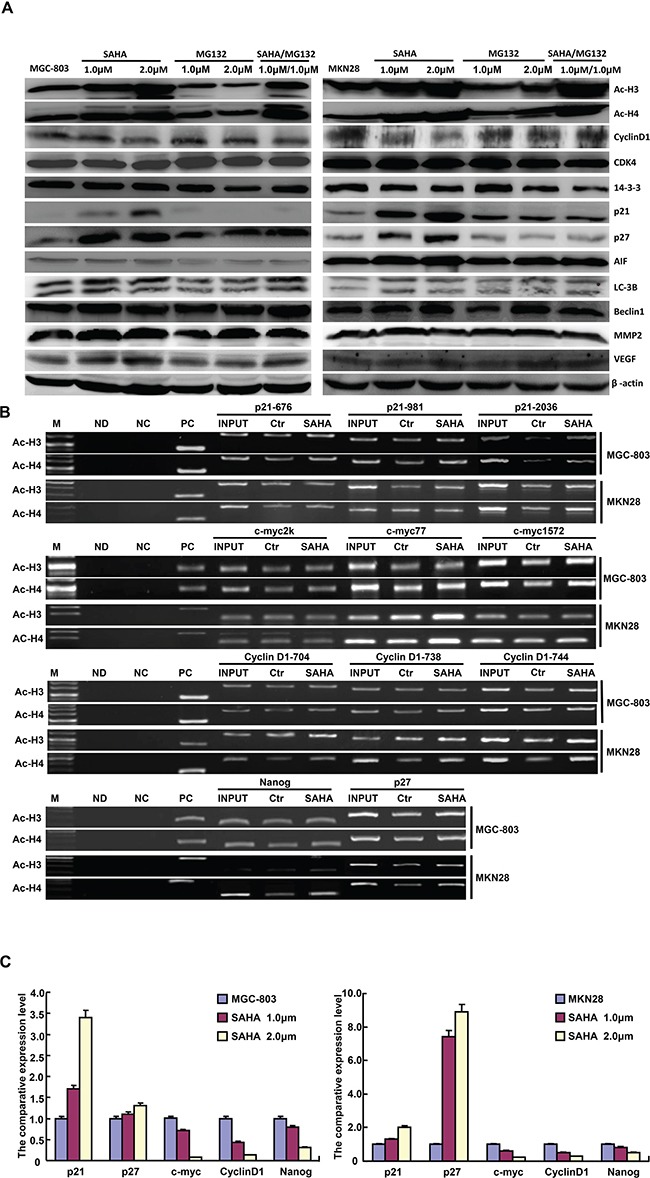
The protein expression profiles and related molecular mechanisms in gastric cancer cells treated with SAHA and MG132 The expression levels of phenotype-related proteins were screened in MGC-803 and MKN28 cells treated with SAHA and/or MG132 by Western blot **A.** ChIP assay showed that acetyl-histone 3 (Ac-H3) and 4 (Ac-H4) might bind to the promoters of p21, Cyclin D1, c-myc, p27 and nanog in MGC-803 and MKN28 cells **B.** SAHA treatment up-regulated the mRNA expression of p21 and p27 in MGC-803 and MKN28 cells, but versa for c- myc, Cyclin D1 and nanog **C.** Note: M, marker; ND: no DNA; NC: negative control using normal mouse IgG ChiP; PC: positive control using anti-Pol II ChiP; INPUT, 2% input; Ctr, control.

After the treatment with SAHA, there was the overexpression of acetyl histone 3 and 4, p21, p27 and LC-3B, Cyclin D1 hypoexpression, but no alteration in CDK4, 14-3-3, AIF, MMP-2, VEGF and Beclin 1 in MGC-803 and MKN-28 cells. MG132 exposure didn't change the expression of acetyl histone 3 and 4, Cyclin D1, CDK4, 14-3-3, p21, p27, AIF, LC-3B Beclin 1, MMP-2 and VEGF (Figure 4A).

ChIP assay showed that acetyl-histone 3 and 4 might bind to the promoters of *p21*, *Cyclin D1*, *c-myc*, *p27* and *nanog* in MGC-803 and MKN28 cells. Acetyl-histone 3 and 4 were recruited to *p21-2036*, *p27*, *c-myc-77*, *Cyclin D1-744*, and *nanog* promoters (Figure 4B), which transcriptionally promoted the expression of *p21* and *p27*, but reduced the expression of *Cyclin D1*, *c-myc* and *nanog*, evidenced by real-time RT-PCR (Figure 4C).

### The association of acetyl-histone 3 and 4 expression with the tumorigenesis and clinicopathological parameters of gastric cancer

**Figure 5:**
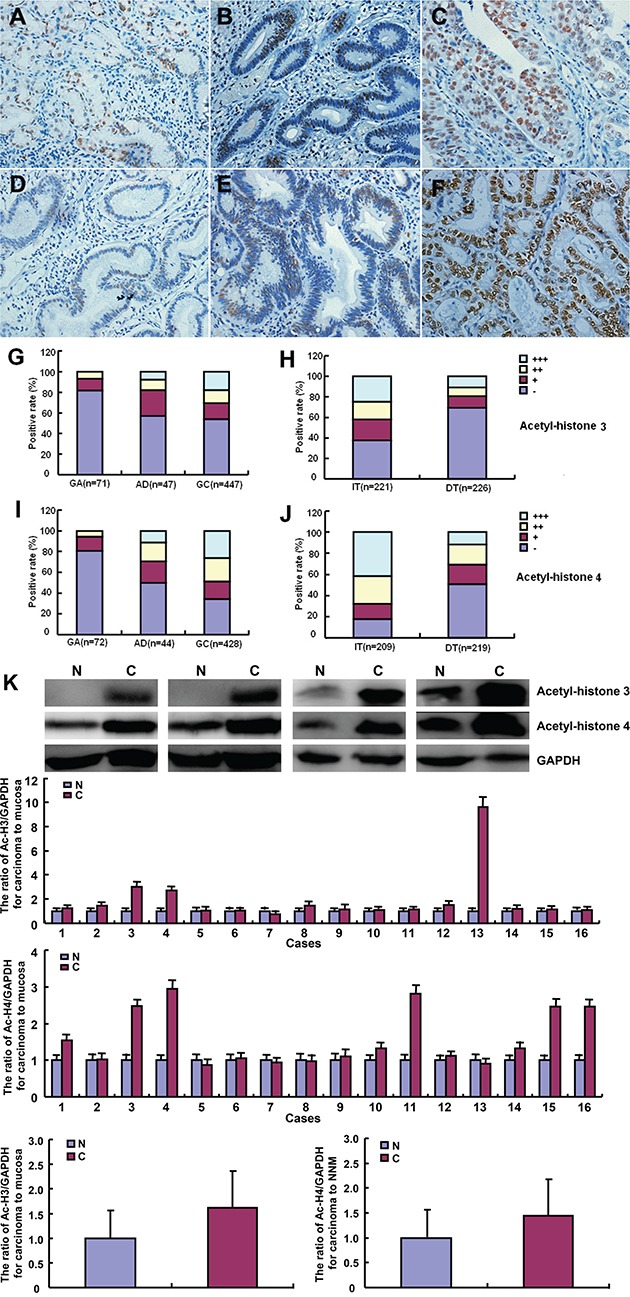
Acetyl-histone 3 and 4 expression in the pathogenesis and aggressiveness of gastric cancers Acetyl-histones 3 and 4 were immunohistochemically expressed in the nuclei of gastric epithelial cells **A, D.**, gastric adenoma **B, E.**, and cancer **C, F.** According to the frequency and density, acetyl-histone 3 or 4 expression was gradually increased during gastritis (GA)- adenoma (AD)-cancer (GC, p<0.05, **G** and **I.**). There was a higher expression of acetyl-histone 3 or 4 in intestinal-type (IT) carcinomas than diffuse-type (DT) carcinomas (p<0.05, **H** and **J.**). The expression levels of both acetyl-histone 3 and 4 were higher in gastric cancer (C) than paired mucosa (N) according to Western blot (p<0.05, **K**).

Immunohistochemically, acetyl-histones 3 and 4 were distributed in the nuclei of gastric epithelial cell, adenoma and cancer (Figure 5A-5F). Statistically, acetyl-histone 3 immunoreactivity was stronger in gastric adenoma and cancer than gastritis (Figure 5G, p<0.01). As shown in Figure 5I, acetyl-histone 4 protein showed higher expression level in gastric cancer than gastric adenoma (p<0.01) and gastritis (p<0.001). Acetyl-histone 4 positivity was stronger in gastric adenoma than gastritis (Figure 5I, p<0.001). In addition, both acetyl- histone 3 and 4 proteins were more expressed in intestinal- than diffuse-type carcinomas (Figure 5H and 5J, p<0.001). As shown in Figure 5K, a higher expression of acetyl-histone 3 and 4 was detectable in gastric cancer than the paired mucosa, evidenced by Western blot (p<0.05).

### The inhibitory effects of SAHA and MG132 treatment on the tumor growth of gastric cancer cells in nude mice

**Figure 6:**
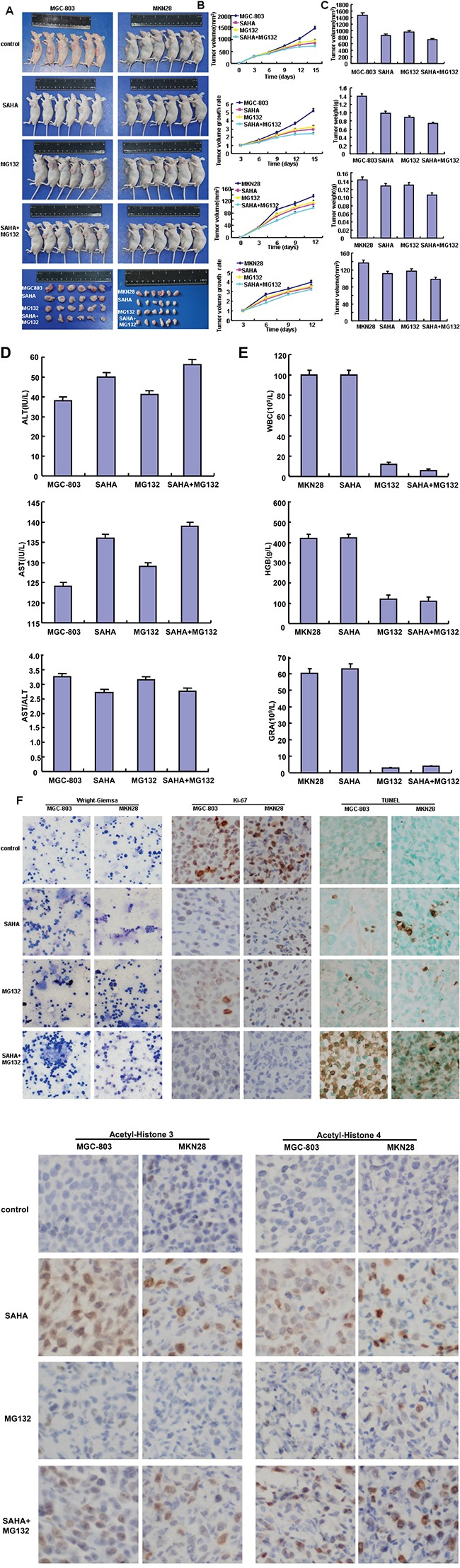
The inhibitory effects of SAHA and MG132 treatment on the tumor growth of gastric cancer cells in xenograft model After MGC-803 and MKN28 were subcutaneously injected into nude mice and treated by SAHA and MG132, the tumor volumes and final weight of xenografts were measured and weighted (**A-C.**, p<0.05). The serum, blood and bone marrow were subjected to the measurement of AST, ALT, blood and marrow cells **D-F.** Finally, the proliferation, histone acetylation and apoptosis of gastric cancer cells were determined by ki-67, acetyl-histones 3 and 4 immunostaining, and TUNEL assay respectively (**F**).

MGC-803 and MKN28 were subcutaneously transplanted into immune- deficient mice. The tumor volumes of xenografts become smaller than the control after the treatment with SAHA or/and MG132 by calculation and weighting respectively (Figure 6A-6C, p<0.05). SAHA or MG132 exposure increased the serum levels of aminoleucine (ALT) and aspartate (AST) aminotransferase in nude model, but only SAHA decreased the AST/ALT ratio (Figure 6D, p<0.05). Furthermore, MG132 remarkably reduced hemoglobin (HGB) level, the number of white blood cell (WBC) and neutrophile granulocyte (GRA), while SAHA didn't (Figure 6E, p<0.05). There appeared synergistic effects of both reagents for above-mentioned six values (Figure 6A-6E). The exposure to SAHA or/and MG132 didn't alter the morphology of bone marrow according to Wright-Giemsa staining, but reduced the proliferation and induced the apoptosis in comparison to the control by ki-67 immunostaining and TUNEL assay respectively (Figure 6F). After treated with SAHA, MGC-803 and MKN28 cells showed a higher expression of acetyl-histone 4 and 3 in the xenograft cancer cells than the control, while not for MG132 (Figure 6F).

## DISCUSSION

The anti-tumor activity of SAHA has been *in vitro* reported in leukemia, mantle cell lymphoma, chondrosarcoma, hepatoma, pancreatic, breast, prostate and colon cancers [7-9]. The cytotoxicity of SAHA was independent of cellular chemoresistance and P-glycoprotein expression [7, 15]. SAHA/parthenolide combination induced GSH depletion, fall in Δψm, release of cytochrome c, Caspase 3 activation and apoptosis by targeting Akt/mTOR/Nrf2 pathway [16]. Xu et al. [17] reported that SAHA exerted significant inhibitory efficiency against cellular survival, proliferation, migration and vasculogenic mimicry of pancreatic cancer cells. The combination of 5-aza-2,-deoxycytidine, cisplatin, or paclitaxel with SAHA inhibited ovarian cancer growth, induced apoptosis, G_2_/M phase arrest and autophagy [18, 19]. Our experimental evidences have shown that SAHA and MG132 reduced cell viability in gastric cancer cells in both dose- and time-dependent manners. In addition, both reagents suppressed the glycolysis and mitochondrial function, induced apoptosis, cell cycle arrest and senescence of gastric cancer cells. It was worth noting that SAHA enhanced cell migration and invasion at a low concentration. Taken together, these findings suggest that SAHA or/and MG132 may inhibit the aggressive phenotypes of gastric cancer cells, but it is essential to maintain a higher serum concentration of SAHA if not combined with MG132.

SAHA is approved for the treatment of cutaneous T-cell lymphoma by the US FDA [20]. Phase I and pharmacodynamic study showed that the combination of SAHA with pelvic palliative radiotherapy, or capecitabine and cisplatin treatment was safe and effective for gastric cancer [21, 22]. It was documented that the combination of SAHA and Cabozantinib resulted in a synergistic induction of cell apoptosis and growth suppression in prostate and lung cancers [23]. Oxaliplatin and SAHA were found to suppress cell survival and growth of gastric [13] and hepatocellular [24] cancer cells by inducing apoptosis or DNA damage. In nude mice, we demonstrated that SAHA and/or MG132 significantly suppressed the tumor growth of gastric cancer cells by decreasing proliferation and increasing apoptosis, in line with the other evidences in prostate [25] and pancreatic [26] cancers. Reportedly, SAHA-mediated inhibition of cell cycle progression and induction of apoptosis were dependent on cell microenvironment and subsequently caused the tumor growth inhibition of colorectal cancer cells [27]. In addition, we found that the administration of SAHA and MG132 damaged the hepatic function, but only MG132 had the cytotoxic effects on peripheral blood cells. Taken together, we suggest that SAHA and MG132 might potentially be employed as chemotherapeutic agents of gastric cancer if the hepatic injury and the killing effects of peripheral blood cells are prevented.

Here, we found that SAHA promoted acetylation of histones 3 and 4 in gastric cancer cells, which were recruited to the promoter of *p21*, *p27*, *c-myc*, *Cyclin D1* and *nanog* for the up-regulated transcription of the former two and the down-regulated expression of the latter three. Both p21cip1/waf1 and p27Kip1 can interact with cyclin-CDK complex and induce G_1_ arrest [28]. Therefore, SAHA might transcriptionally up-regulate the expression of p21 and p27 at both protein and mRNA level to cause G_1_ arrest of gastric cancer cells, which SAHA- induced Cyclin D1 hypoexpression was also responsible for. It was reported that SAHA exposure up-regulated the expression of Cyclin D1 in colon cancer cells [29] and mantle cell lymphoma cells [30]. SAHA was demonstrated to reverse chemoresistance in head and neck cancer cells by targeting cancer stem cells via the down-regulation of nanog [31], in line with our result. Wang et al. [32] found that K-ras confered SAHA resistance by up-regulating c-myc expression. Although SAHA decreased levels of c-myc in pancreatic cancer cells [26], we found that acetyl histones bound to the promoter of c-myc and suppressed its transcription, finally to reverse the aggressive phenotypes of gastric cancer cells. Additionally, our results also showed no difference in the expression of VEGF and MMP-2 in gastric cancer cells treated by SAHA or/and MG132, suggesting that their regulatory effects on migration and invasion were independent of both molecules. SAHA increased LC-3B expression, but didn't alter the expression level of Beclin 1, suggesting that the inducing effect of SAHA on autophagy was independent of Beclin 1 and didn't belong to classic pathway of autophagy.

In xenograft model and cell experiment, we found that SAHA up-regulated histone acetylation, supporting the opinion that histones 3 and 4 are the potent targets of SAHA. According to the data of immunohistochemistry and Western blot, we for the first time found that expression of both acetyl-histones 3 and 4 were significantly higher in gastric cancer than in adenoma and gastritis, indicating that their overexpression may be a reactive response and might increase SAHA sensitivity during gastric carcinogenesis. Reportedly, the expression of histones 3 and 4 would be potential markers to monitor the efficacy of SAHA as described in peripheral blood mononuclear cells [33]. Opposite to previous reports about ovarian cancer [7] and renal cell carcinoma [34], both proteins were positively linked to the differentiation degree of gastric cancer, indicating that they might underlie the molecular mechanisms of the differentiation of gastric cancer.

In conclusion, SAHA and MG132 *in vivo* and *vitro* have the synergistic effects of cytotoxicity and aggressive phenotypes reversing in gastric cancer cells by inducing apoptosis, suppressing proliferation, growth, migration, or invasion. SAHA of low concentration might promote migration and invasion of gastric cancer cells. SAHA may increase the expression of acetyl-histone 3 and 4 and thereby upregulate or down-regulate the mRNA expression of downstream genes, including *Cyclin D1*, *p21*, *p27*, *c-myc* and *nanog*. The histone acetylation may be positively linked to the tumorigenesis and differentiation of gastric cancer. Therefore, SAHA or/and MG132 could potentially be employed as chemotherapeutic drugs in clinical practice.

## MATERIALS AND METHODS

### Cell culture

Gastric cancer cell lines, MGC-803 (poorly-differentiated adenocarcinoma) and MKN28 (well-differentiated adenocarcinoma) were purchased from the ATCC (Manassas, VA, USA). The cells were maintained in RPMI-1640 media, supplemented with 10% fetal bovine serum (FBS), 100 U/mL penicillin and 100 μg/mL streptomycin in a humidified atmosphere of 5% CO_2_ at 37°C. All cells were harvested by centrifugation, rinsed with phosphate buffered saline (PBS), and subjected to total protein and RNA extraction. We exposed cells to SAHA and MG132 for the following experiments.

### Proliferation assay

Cell counting Kit-8 (CCK-8; Dojindo, Kumamoto, Japan) was employed to determine the number of viable cells. In brief, 2.5 × 10^3^ cells/well were seeded into 96-well plates and allowed to adhere. At specified time points, 10 μL CCK-8 solution was added to each well and the plates were incubated for a further 3h. The number of viable cells was counted by measuring the absorbance at 450 nm.

### Cell cycle analysis

The cells were detached by trypsinization, collected, washed twice with PBS and fixed in 10 mL ice-cold ethanol for at least 2h. The cells were washed twice with PBS again and incubated with 1 mL RNase (0.25 mg/mL) at 37°C for 1h. The cells were pelleted, resuspended in propidium iodide (PI, 50 μg/mL), and incubated in the dark for 30 min. Cell cycle analysis was performed by analysis of PI staining by flow cytometry.

### Apoptosis assay

Flow cytometry was performed following FITC-labeled Annexin V and PI staining (*KeyGEN* Biotech) to detect phosphatidylserine externalization as an endpoint indicator of apoptosis. In brief, cells were washed with PBS, resuspended in 1 × Binding Buffer, and then incubated with 5 μL FITC-Annexin V and 5 μL PI. Samples were gently vortexed and incubated for 15min in the dark, then 400 μL 1 × Binding Buffer was added to each tube. Flow cytometry was performed within 1 h of incubation.

### Wound healing assay

1.0 × 10^6^ cells were seeded in 6-well culture plates and scratched with a pipette tip when reaching 80% confluence. Cells were washed three times with PBS, and cultured in FBS-free medium. Cells were photographed and the scratch area was measured using Image J software.

### Cell invasion assay

2.5 × 10^5^ cells were resuspended in serum-free RPMI-1640 and seeded into the top chamber of matrigel-coated transwell inserts. The lower compartment of the chamber contained 10% FBS as a chemoattractant. After incubation for 24 h, the cells on the upper surface of the membrane were wiped away, and the cells on the lower surface of the membrane were washed with PBS, fixed in methanol and stained with Giemsa dye to quantify the extent of invasion.

### Immunofluorescence

Cells were grown on glass coverslips, fixed with PBS containing 4% formaldehyde for 10 min, and permeabilized with 0.2% Triton X-100 in PBS for 10. After washing with PBS, the cells were incubated overnight at 4°C with Alexa Fluor 594 Phalloidin (Invitrogen) to indicate the lamellipodia. Nuclei were stained with 1 μg/mL DAPI (Sigma-Aldrich) for 15 min at 37°C. The coverslips were then mounted with SlowFade Gold Antifade Reagent (Invitrogen) and observed under a confocal laser microscope (Olympus, Tokyo, Japan).

### β-galactosidase staining

β-galactosidase staining kit (Beyotime, China) was used to indicate the senescence. Cells (5×10^5^) were seeded in 6-well dishes, incubated for 2 days. All cells were washed two times with PBS and fixed with 4% paraformaldehyde for 15 min at room temperature. Then the cells were incubated overnight at 37 °C with the working solution containing 0.05 mg/mL X-gal. Finally, the cells were examined under a light inverted microscope.

### Metabolism assays

Oxygen consumption rates and extracellular acidification rates were measured in XF media (nonbuffered RPMI 1640 containing either 10mM or 25 mM glucose or galactose, 2 mM L-glutamine, and 1 mM sodium pyruvate) under basal conditions and in response to mitochondrial inhibitors, 1 mM oligomycin and/or 100 nM rotenone + 1 mM antimycin A (Sigma) on the XF-24 or XF-96 Extracellular Flux Analyzers (Seahorse Bioscience). ATP measurements were performed with the ATP determination kit (Invitrogen) and glucose concentrations were measured with a glucose assay kit (Eton Bioscience Inc.).

### Selection of patient samples

Samples of gastric cancer (n=447), adenoma (n=47) and gastritis (n=72) were collected from patients undergoing surgical resection between January 2003 and December 2011 at our hospital. The average age of the patients at surgery was 51.6 years (range 20–81 years). Sixteen cases of fresh gastric cancer and matched mucosa were also sampled from our hospital. None of the patients had undergone chemotherapy, radiotherapy or adjuvant treatment prior to surgery. Informed written consent was obtained from all participants and the study was approved by our University Ethics Committee.

### Pathology and tissue microarray (TMA) analysis

Tumor histology was determined according to Lauren's classification system [35]. TMA was established as reported previously [35]. Consecutive 4 μm sections were incised from the recipient block and transferred to poly-lysine-coated glass slides.

### Western blot analysis

After protein, denatured proteins were separated by sodium dodecyl sulfate- polyacrylamide gel electrophoresis on acrylamide gel, and transferred to Hybond membranes. The membranes were blocked overnight in 5% skim milk in TBST. For immunoblotting, the membranes were incubated with the primary antibody (Table 3), rinsed with TBST, and incubated with IgG antibodies conjugated to horseradish peroxidase (HRP; Dako). Bands were visualized using X-ray film by ECL-Plus detection reagents. Densitometric quantification of acetyl-histone 3 and 4 protein expression in gastric samples was performed using Image J software, with GAPDH as a control.

### Chromatin immunoprecipitation (ChiP)

ChiP assays were performed using Magna ChipTM G kit (Upstate) according to manufacturer's instructions. The primer sequences were targeted to the gene of *c-myc*, *Cyclin D1*, *p21*, *p27*, and *nanog* (Table 1). PCR amplification was performed in 20μL mixtures and amplicons were separated in agarose gel.

**Table 1:** The primers used for PCR, followed by CHIP

Name	Primer's sequence	AT (°C)	Product size(bp)	Extension time(s)
c-myc-2k	F:5′-TCACGTTTGCCATTACCGGTTC −3′ R:5′-TTTCAGGTTGGCTGCA G A AGGT-3′	58	171	30
c-myc-77	F:5′-CAGGGCTTCTCAGAGGCTTGG-3′ R:5′-CTGCTCGCCCGGCTCTTCC ACC-3′	58	162	30
c-myc-1572	F:5′-CAGATCAGCAACAACCG AAA-3′ R:5′-GGCCTTTTCATTGTTTTCCA-3′	58	167	30
Cyclin d704	F:5′-TGAAAATGAAAGAAGATGCAGTCG-3′ R:5′-CTGTAGTCCGGTTTTCATAGAAATGC-3′	57	328	30
Cyclin d738	F:5′-GTCCTACTTCAAATGTGTGCAGAAGG-3′ R:5′-CTCCCACGAAACGCTACTTCTAGC-3′	57	290	30
Cyclin d744	F:5′-CCCAGTTACTGTCGTTATCTCTCATC-3′ R:5′-ATCCCTTTTGTAGCATCCCAAGAG-3′	57	294	30
p21-676	F:5′-CCCGGAAGCATGTGACAATC-3′ R:5′-CAGCACTGTTAGAATGAGCC-3′	56	354	30
p21-981	F:5′-GGAGGCAAAAGTCCTGTGTTC-3′ R:5′-GGAAGGAGGGAATTGGAGAG-3′	56	306	30
p21-2036	F:5′-GGAGTCAGATTCTGTGTGTG-3′ R:5′-CCTCTGCTTTCAGGCATTTC-3′	56	368	30
P27	F:5′-CTGTCACATTCTGGAGCGTA-3′ R:5′-AGTGGATCTTCAACTGCCTC-3′	60	230	30
Nanog	F: 5′-CAAAGGCAAACAACCCACTT-3′ R: 5′-TCTGCTGGAGGCTGAGGTAT-3′	60	95	30

### Real-time reverse transcriptase–polymerase chain reaction (real-time RT-PCR)

Total RNA was extracted from the gastric cancer cell lines using Trizol (Takara) Real-time RT-PCR was performed from 2 μg of total RNA using AMV reverse transcriptase and random primers (Takara). PCR primers were designed according to the sequences in GenBank and are listed in Table 2. Amplification of cDNA was performed using the SYBR Premix Ex Taq II kit (Takara), using *GAPDH* as an internal control.

**Table 2:** The primers used for real-time RT-PCR

Name	Primer's sequence		AT (°C)	Product size(bp)	Extension time(s)
p21	F: 5′-ACTGTCTTGTACCCTTGTGCC-3′ R: 5′-AAATCTGTCATGCTGGTCTGC-3′	XM_003950827 572-679	55	108	60
p27	F: 5′-GGCTCCGGCTAACTCTGA-3′ R: 5′-TTCTTCTGTTCTGTTGGCTCTT-3′	XM_522347 1081-1237	55	157	60
c-myc	F: 5′-AGCGACTCTGAGGAGGAACA-3′ R: 5′-TCCAGCAGAAGGTGATCCA-3′	X00676 1318-1425	55	108	60
Cyclin D1	F: 5′-TGCCACAGATGTGAAGTTCATT-3′ R: 5′-CAGTCCGGGTCACACTTGAT-3′	NG_000002 776-937	55	162	60
Nanog	F: 5′-CAAAGGCAAACAACCCACTT-3′ R: 5′TCTGCTGGAGGCTGAGGTAT-3′	XM_011520852.1 360-517	55	158	60
GAPDH	F: 5′-CAATGACCCCTTCATTGACC-3′ R: 5′- TGGAAGATGGTGATGGGATT-3′	NM_ 002046.3 201-335	55	135	60

**Table 3:** The antibodies used for Western blot, CHIP and immunohistochemistry

Num	Antibody	Species	Dilution	Company	Code number
1	Ac-Histone 3 (Lys 9/14)	rabbit	1:1000	santa cruz	sc-8655-R
2	Ac-Histone 4 (Lys 8)	rabbit	1:1000	santa cruz	sc-8660-R
3	Cyclin D1(H-295)	rabbit	1:500	santa cruz	sc-753
4	Cdk4 (C-22)	rabbit	1:500	santa cruz	sc-260
5	14-3-3 (H-8)	mouse	1:500	santa cruz	sc-1657
6	p21 (F-5)	mouse	1:500	santa cruz	sc-6246
7	p27 (N-20)	mouse	1:500	santa cruz	sc-527
8	AIF (E-1)	mouse	1:700	santa cruz	sc-13116
9	LC-3B	rabbit	1:1000	wanleibio	wl01506
10	Beclin 1	rabbit	1:2000	abcam	Ab51031
11	MMP2	rabbit	1:1000	wanleibio	wl0137
12	VEGF	rabbit	1:500	santa cruz	sc-152
13	β-actin(C4)	mouse	1:2000	santa cruz	sc-47778

### Xenograft models

Locally bred female Balb/c nude (nu/nu) mice were used for implantation at the age of 6-8 weeks. They were maintained under specific pathogen-free conditions. Housing and all procedures were performed according to protocols approved by the Committee for Animal Experiments Guidelines on Animal Welfare of Jinzhou Medical University. Subcutaneous xenografts were established by injection of 1× 10^6^ cells per mouse to axilla (n=10 mice /group). From tumor diameter reached 8mm, we began to intraperitoneally inject 20 mg/kg SAHA, 2 mg/kg MG132 and 20 mg/kg SAHA + 2 mg/kg MG132 into mice from 8^th^, 10^th^, and 12^th^ day of cell injection. After anesthetization, the mice were photographed, and sacrificed. For each tumor, measurements were made using calipers, and tumor volumes were calculated as follows: width^2^× length ×0.52. The tumors were subsequently fixed in 4% paraformaldehyde, and then embedded in paraffin for the preparation of blocks.

### The measurement of serum enzyme, blood and bone marrow cells

The peripheral blood of nude mice was collected from abdominal vein, kept into a disposable venous blood sample collection vessel, and centrifuged at 4000 rpm/min for 5 min. Afterwards, the supernatant was analyzed for alanine aminatransferase, aspartate aminotransferase, AST/ALT ratio by automatic biochemical analyzer (Hitachi 7600). And then, another peripheral blood samples were kept in BD vacutainer including EDTA-K_2_. The blood cells indexes, such as total white blood cell count, neutrophil and hemoglobin, were tested by automated hematology analyzer with five classifications (Sysmex XS-500i). Finally, following the separation of femurs, the bone marrow cells were harvested from epiphysis of femurs and microscope slides were prepared. The Giemsa-Wright staining method was used to observe the bone marrow cells morphology and the proportion of the cells under biological microscope.

### Immunohistochemistry

Consecutive sections of tissue samples were deparaffinized with xylene, rehydrated with alcohol, and subjected to the immunohistochemical staining of intermittent wave irradiation as previously described [36]. The rabbit anti-acetyl-histone-3 (Lys 9/14), anti-acetyl-histone 4 (Lys 8), and anti-ki-67 antibody and anti-rabbit antibodies conjugated to HRP were purchased from Santa Cruz Biotechnology and Dako respectively. Negative controls were prepared by omitting the primary antibody.

Two independent observers (LH and ZHC) randomly selected and counted 100 cells from five representative fields from each section. Any discrepancies were checked by both observers until a consensus was reached. Positive expression was graded as follows: 0 = negative; 1 = 1%–50%; 2 = 50%–74%; 3 ≥ 75%. The staining intensity was graded as follows: 1 = weak; 2 = intermediate; 3 = strong. The two grades were multiplied to obtain a final score: – = 0; + = 1–2; ++ = 3–5; +++ = 6–9).

### Terminal digoxigenin-labeled dUTP nick-end labeling (TUNEL)

Cell apoptosis was assessed using TUNEL, a method that is based on the specific binding O-TdT to the 3-OH ends of DNA. For this purpose, ApopTag Plus Peroxidase *In Situ* Apoptosis Detection Kit (Chemicon) was employed according to the recommendation. Omission of the working strength TdT enzyme was considered as a negative control.

### Statistical analysis

Statistical evaluation was performed using Spearman's rank correlation coefficient to analyze ranked data, and Mann-Whitney U to differentiate the means of different groups. A p-value < 0.05 was considered statistically significant. SPSS 10.0 software was employed to analyze all data.

## References

[1] Mottamal M, Zheng S, Huang TL, Wang G (2015). Histone deacetylase inhibitors in clinical studies as templates for new anticancer agents. Molecules.

[2] Tan S, Liu ZP (2015). Natural products as zinc-dependent histone deacetylase inhibitors. Chem Med Chem.

[3] Marks PA (2007). Discovery and development of SAHA as an anticancer agent. Oncogene.

[4] Codd R, Braich N, Liu J, Soe CZ, Pakchung AA (2009). Zn(II)-dependent histone deacetylase inhibitors: suberoylanilide hydroxamic acid and trichostatin A. Int J Biochem Cell Biol.

[5] Zheng L, Fu Y, Zhuang L, Gai R, Ma J, Lou J, Zhu H, He Q, Yang B (2014). Simultaneous NF-κB inhibition and E-cadherin upregulation mediate mutually synergistic anticancer activity of celastrol and SAHA in vitro and in vivo. Int J Cancer.

[6] Butler LM, Zhou X, Xu WS, Scher HI, Rifkind RA, Marks PA, Richon VM (2002). The histone deacetylase inhibitor SAHA arrests cancer cell growth, up-regulates thioredoxin-binding protein-2, and down-regulates thioredoxin. Proc Natl Acad Sci U S A.

[7] Chen S, Zhao Y, Gou WF, Zhao S, Takano Y, Zheng HC (2013). The anti-tumor effects and molecular mechanisms of suberoylanilide hydroxamic acid (SAHA) on the aggressive phenotypes of ovarian carcinoma cells. PLoS One.

[8] You BR, Park WH (2014). Suberoylanilide hydroxamic acid-induced HeLa cell death is closely correlated with oxidative stress and thioredoxin 1 levels. Int J Oncol.

[9] Ding L, Zhang Z, Liang G, Yao Z, Wu H, Wang B, Zhang J, Tariq M, Ying M, Yang B (2015). SAHA triggered MET activation contributes to SAHA tolerance in solid cancer cells. Cancer Lett.

[10] Liu Z, Tong Y, Liu Y, Liu H, Li C, Zhao Y, Zhang Y (2014). Effects of suberoylanilide hydroxamic acid (SAHA) combined with paclitaxel (PTX) on paclitaxel-resistant ovarian cancer cells and insights into the underlying mechanisms. Cancer Cell Int.

[11] Peters MD (2014). Postsurgical chemotherapy vs. surgery alone for resectable gastric cancer. Am J Nurs.

[12] Yoo C, Ryu MH, Na YS, Ryoo BY, Lee CW, Maeng J, Kim SY, Koo DH, Park I, Kang YK (2014). Phase I and pharmacodynamic study of vorinostat combined with capecitabine and cisplatin as first-line chemotherapy in advanced gastric cancer. Invest New Drugs.

[13] Zhou C, Ji J, Shi M, Yang L, Yu Y, Liu B, Zhu Z, Zhang J (2014). Suberoylanilide hydroxamic acid enhances the antitumor activity of oxaliplatin by reversing the oxaliplatin-induced Src activation in gastric cancer cells. Mol Med Rep.

[14] Huang C, Ida H, Ito K, Zhang H, Ito Y (2007). Contribution of reactivated RUNX3 to inhibition of gastric cancer cell growth following suberoylanilide hydroxamic acid (vorinostat) treatment. Biochem Pharmacol.

[15] Ruefli AA, Bernhard D, Tainton KM, Kofler R, Smyth MJ, Johnstone RW (2002). Suberoylanilide hydroxamic acid (SAHA) overcomes multidrug resistance and induces cell death in P-glycoprotein-expressing cells. Int J Cancer.

[16] Carlisi D, Lauricella M, D'Anneo A, Buttitta G, Emanuele S, di Fiore R, Martinez R, Rolfo C, Vento R, Tesoriere G (2015). The Synergistic Effect of SAHA and Parthenolide in MDA-MB231 Breast Cancer Cells. J Cell Physiol.

[17] Xu XD, Yang L, Zheng LY, Pan YY, Cao ZF, Zhang ZQ, Zhou QS, Yang B, Cao C (2014). Suberoylanilide hydroxamic acid, an inhibitor of histone deacetylase, suppresses vasculogenic mimicry and proliferation of highly aggressive pancreatic cancer PaTu8988 cells. BMC Cancer.

[18] Angelucci A, Mari M, Millimaggi D, Giusti I, Carta G, Bologna M, Dolo V (2010). Suberoylanilide hydroxamic acid partly reverses resistance to paclitaxel in human ovarian cancer cell lines. Gynecol Oncol.

[19] Dietrich CS, Greenberg VL, DeSimone CP, Modesitt SC, van Nagell JR, Craven R, Zimmer SG (2010). Suberoylanilide hydroxamic acid (SAHA) potentiates paclitaxel-induced apoptosis in ovarian cancer cell lines. Gynecol Oncol.

[20] Duvic M, Talpur R, Ni X, Zhang C, Hazarika P, Kelly C, Chiao JH, Reilly JF, Ricker JL, Richon VM, Frankel SR (2007). Phase 2 trial of oral vorinostat (suberoylanilide hydroxamic acid, SAHA) for refractory cutaneous T-cell lymphoma (CTCL). Blood.

[21] Ree AH, Dueland S, Folkvord S, Hole KH, Seierstad T, Johansen M, Abrahamsen TW, Flatmark K (2010). Vorinostat, a histone deacetylase inhibitor, combined with pelvic palliative radiotherapy for gastrointestinal carcinoma: the Pelvic Radiation and Vorinostat (PRAVO) phase 1 study. Lancet Oncol.

[22] Kelly WK, O'Connor OA, Krug LM, Chiao JH, Heaney M, Curley T, MacGregore-Cortelli B, Tong W, Secrist JP, Schwartz L, Richardson S, Chu E, Olgac S, Marks PA, Scher H, Richon VM (2005). Phase I study of an oral histone deacetylase inhibitor, suberoylanilide hydroxamic acid, in patients with advanced cancer. J Clin Oncol.

[23] Ding L, Zhang Z, Liang G, Yao Z, Wu H, Wang B, Zhang J, Tariq M, Ying M, Yang B (2015). SAHA triggered MET activation contributes to SAHA tolerance in solid cancer cells. Cancer Lett.

[24] Hsu FT, Liu YC, Chiang IT, Liu RS, Wang HE1, Lin WJ4, Hwang JJ (2014). Sorafenib increases efficacy of vorinostat against human hepatocellular carcinoma through transduction inhibition of vorinostat-induced ERK/NF-κB signaling. Int J Oncol.

[25] Wang L, Zou X, Berger AD, Twiss C, Peng Y, Li Y, Chiu J, Guo H, Satagopan J, Wilton A, Gerald W, Basch R, Wang Z, Osman I, Lee P (2009). Increased expression of histone deacetylaces (HDACs) and inhibition of prostate cancer growth and invasion by HDAC inhibitor SAHA. Am J Transl Res.

[26] Kumagai T, Wakimoto N, Yin D, Gery S, Kawamata N, Takai N, Komatsu N, Chumakov A, Imai Y, Koeffler HP (2007). Histone deacetylase inhibitor, suberoylanilide hydroxamic acid (Vorinostat, SAHA) profoundly inhibits the growth of human pancreatic cancer cells. Int J Cancer.

[27] Lobjois V, Frongia C, Jozan S, Truchet I, Valette A (2009). Cell cycle and apoptotic effects of SAHA are regulated by the cellular microenvironment in HCT116 multicellular tumour spheroids. Eur J Cancer.

[28] Yoon MK, Mitrea DM, Ou L, Kriwacki RW (2012). Cell cycle regulation by the intrinsically disordered proteins p21 and p27. Biochem Soc Trans.

[29] Jin JS, Tsao TY, Sun PC, Yu CP, Tzao C (2012). SAHA Inhibits the growth of colon tumors by decreasing histone deacetylase and the expression of Cyclin D1 and survivin. Pathol Oncol Res.

[30] Kawamata N, Chen J, Koeffler HP (2007). Suberoylanilide hydroxamic acid (SAHA; vorinostat) suppresses translation of cyclin D1 in mantle cell lymphoma cells. Blood.

[31] Kumar B, Yadav A, Lang JC, Teknos TN, Kumar P (2015). Suberoylanilide hydroxamic acid (SAHA) reverses chemoresistance in head and neck cancer cells by targeting cancer stem cells via the downregulation of nanog. Genes Cancer.

[32] Wang Q, Tan R, Zhu X, Zhang Y, Tan Z, Su B, Li Y (2016). Oncogenic K-ras confers SAHA resistance by up-regulating HDAC6 and c-myc expression. Oncotarget.

[33] Liu L, Detering JC, Milde T, Haefeli WE, Witt O, Burhenne J (2014). Quantification of vorinostat and its main metabolites in plasma and intracellular vorinostat in PBMCs by liquid chromatography coupled to tandem mass spectrometry and its relation to histone deacetylase activity in human blood. J Chromatogr B Analyt Technol Biomed Life Sci.

[34] Minardi D, Lucarini G, Filosa A, Milanese G, Zizzi A, Di Primio R, Montironi R, Muzzonigro G (2009). Prognostic role of global DNA-methylation and histone acetylation in pT1a clear cell renal carcinoma in partial nephrectomy specimens. J Cell Mol Med.

[35] Zheng HC, Li XH, Hara T, Masuda S, Yang XH, Guan YF, Takano Y (2008). Mixed-type gastric carcinomas exhibit more aggressive features and indicate the histogenesis of carcinomas. Virchows Arch.

[36] Kumada T, Tsuneyama K, Hatta H, Ishizawa S, Takano Y (2004). Improved 1-h rapid immunostaining method using intermittent microwave irradiation: practicability based on 5 years application in Toyama Medical and Pharmaceutical University Hospital. Mod Pathol.

